# Exposure to Phthalates and Phenols during Pregnancy and Offspring Size at Birth

**DOI:** 10.1289/ehp.1103634

**Published:** 2011-09-07

**Authors:** Claire Philippat, Marion Mortamais, Cécile Chevrier, Claire Petit, Antonia M. Calafat, Xiaoyun Ye, Manori J. Silva, Christian Brambilla, Isabelle Pin, Marie-Aline Charles, Sylvaine Cordier, Rémy Slama

**Affiliations:** 1 Institut National de la Santé et de la Recherche Médicale (INSERM), Institut Albert Bonniot (U823), Team of Environmental Epidemiology Applied to Reproduction and Respiratory Health, Grenoble, France; 2 Grenoble University, Institut Albert Bonniot, Grenoble, France; 3 INSERM, U1061, Montpellier, France; 4 INSERM, U625, University of Rennes I, Institut fédératif de recherche 140 (IFR140); Rennes, France; 5 Centers for Disease Control and Prevention, Atlanta, Georgia, USA; 6 INSERM and Grenoble University, Institut Albert Bonniot (U823), Molecular Basis of Lung Cancer Progression, Grenoble, France; 7 Service de Pédiatrie, Centre Hospitalier Universitaire de Grenoble, France; 8 INSERM, U1018, Centre de recherche en Épidémiologie et Santé des Populations (CESP), Team Epidemiology of Diabetes, Obesity and Renal Disease: Lifelong Approach, Villejuif, France

**Keywords:** birth outcomes, fetal growth, phenols, phthalates, pregnancy exposure, urinary biomarkers

## Abstract

Background: Data concerning the effects of prenatal exposures to phthalates and phenols on fetal growth are limited in humans. Previous findings suggest possible effects of some phenols on male birth weight.

Objective: Our aim was to assess the relationships between prenatal exposures to phthalates and phenols and fetal growth among male newborns.

Methods: We conducted a case–control study on male malformations of the genitalia nested in two French mother–child cohorts with recruitment between 2002 and 2006. We measured, in maternal urinary samples collected between 6 and 30 gestational weeks, the concentrations (micrograms per liter) of 9 phenol (*n* = 191 pregnant women) and 11 phthalate metabolites (*n* = 287). Weight, length, and head circumference at birth were collected from maternity records. Statistical analyses were corrected for the oversampling of malformation cases.

Results: Adjusted birth weight decreased by 77 g [95% confidence interval (CI): –129, –25] and by 49 g (95% CI: –86, –13) in association with a 1-unit increase in ln-transformed 2,4-dichlorophenol (DCP) and 2,5-DCP urinary concentrations, respectively. Benzophenone-3 (BP3) ln-transformed concentrations were positively associated with weight (26 g; 95% CI: –2, 54) and head circumference at birth (0.1 cm; 95% CI: 0.0, 0.2). Head circumference increased by 0.3 cm (95% CI: 0.0, 0.7) in association with a 1-unit increase in ln-transformed BPA concentration. For phthalate metabolites there was no evidence of monotonic associations with birth weight.

Conclusions: Consistent with findings of a previous study, we observed evidence of an inverse association of 2,5-DCP and a positive association of BP3 with male birth weight.

Diesters of phthalic acid (phthalates) and phenols are found in many consumer products. Low molecular weight (MW) phthalates (MW < 250 g/mol) are used in personal care products (perfumes, cosmetics) or as coating for pharmaceutical products. High MW phthalates (MW > 250 g/mol) tend to be used in polyvinylchloride floor and wall covering, food packaging, and medical devices (Calafat et al. 2006; Hauser and Calafat 2005). Phenols are used in food packaging [bisphenol A, (BPA)], polycarbonates (BPA), cosmetics (parabens), soap [triclosan (TCS)], and sunscreen [benzophenone-3 (BP3)] (Calafat et al. 2008). Precursors of dichlorophenols (DCPs) are used as intermediates in the production of several herbicides or insecticides (Agency for Toxic Substances and Disease Registry 2006).

Widespread exposure to phthalates and phenols has been documented for pregnant women in several industrialized countries (Adibi et al. 2008; Braun et al. 2011; Cantonwine et al. 2010; Wolff et al. 2008; Ye et al. 2008, 2009). Some of these compounds can cross the placenta in humans (Balakrishnan et al. 2010; Mose et al. 2007), and phthalates have been detected in cord blood (Latini et al. 2003), amniotic fluid (Huang et al. 2009; Silva et al. 2004), and meconium (Zhang et al. 2009).

Little is known about the consequences of prenatal exposures to phthalates and phenols on fetal growth. An American cohort study of 404 mother–infant pairs reported an inverse association for 2,5-DCP maternal urinary concentrations and a positive association for BP3 urinary concentrations with birth weight in male but not in female newborns (Wolff et al. 2008).

Our aim was to study the relationships between prenatal exposures to phthalates and phenols and weight, length, and head circumference at birth among male newborns.

## Population and Methods

*Study population.* We conducted a case–control study of male malformations of the genitalia nested in the EDEN (Etude des Déterminants pré et post natals du développement et de la santé de l’Enfant) and PELAGIE mother–child cohorts. These cohorts are described elsewhere (Drouillet et al. 2009; Garlantezec et al. 2009). Briefly, the EDEN cohort consists of 2,002 pregnant women recruited before the end of the 28th gestational week from April 2003 through March 2006 in the obstetrical departments of the University Hospitals of Nancy and Poitiers, France. The PELAGIE cohort consists of 3,421 pregnant women enrolled before 19 weeks of gestation from April 2002 through February 2006 in three districts of Brittany: Ille et Vilaine, Finistère, and Côtes d’Armor, France. The present study includes all of the male newborns with undescended testis or hypospadias (identified at birth by pediatricians; *n* = 48 in EDEN and 24 in PELAGIE). In addition, three male newborns without congenital malformation of the genitalia (controls) were matched to each case by recruitment center, date of recruitment (± 6 months), day of week (weekend yes/no), and gestational week when the maternal urine sample was collected, for a total of 288 mother–newborn pairs (72 cases and 216 controls). Participants provided informed consent for data and biological sample collection for themselves and their offspring. These cohorts received the approvals of the appropriate ethical committees. The involvement of the Centers for Disease Control and Prevention (CDC) laboratory was limited and determined not to constitute engagement in human subjects research.

*Outcomes.* We extracted from hospital maternity records weight, length, and head circumference assessed at birth.

*Exposure assessment.* Urine was collected between 6 and 19 gestational weeks in the PELAGIE cohort and between 24 and 30 gestational weeks in the EDEN cohort. Assessment of biomarker and creatinine concentrations in maternal urine was done by the National Center for Environmental Health laboratory at the CDC in Atlanta, Georgia, USA (Silva et al. 2007; Ye et al. 2005). We measured the urinary concentrations of 11 phthalate metabolites in samples from all mothers (*n* = 287; 72 cases and 215 controls because the vial of one control broke during transport to the CDC laboratory). Nine phenols were measured in urine samples collected from mothers in the EDEN cohort only (*n* = 191; 48 cases and 143 controls) (phenols could not be measured in the PELAGIE cohort because a preservative added to the samples interferes with the assay used).

Molar concentrations of four metabolites of di(2-ethylhexyl) phthalate (DEHP) [mono(2-ethylhexyl) phthalate (MEHP); mono(2-ethyl-5-hydroxyhexyl) phthalate (MEHHP); mono(2-ethyl-5-oxohexyl) phthalate (MEOHP); and mono(2-ethyl-5-carboxypentyl) phthalate (MECPP)] were summed as total DEHP. Concentrations of phthalate metabolites of MW > 250 g/mol and of MW < 250 g/mol were summed as total of high MW phthalates (ΣHMW) and of low MW phthalates (ΣLMW), respectively. Total paraben concentration (ΣPB) was calculated by summing methyl paraben (MP), ethyl paraben (EP), propyl paraben (PP), and butyl paraben (BP) molar concentrations. Monoethyl phthalate (MEP) and monobenzyl phthalate (MBzP) concentrations have been corrected [multiplied by 0.66 (MEP) and 0.72 (MBzP)] because the analytical standards used were of inadequate purity (Calafat AM, personal communication).

*Statistical analyses.* We replaced concentrations below the limit of detection (LOD) by LOD/_√_^–^2. We corrected the overrepresentation of congenital abnormalities, induced by the case–control design, using a reweighting approach. We used center-specific weights corresponding to the inverse of the inclusion probability of controls, so as to give cases and controls the same relative weight as in the original cohorts (Richardson et al. 2007). Sensitivity analyses restricted to controls were also conducted.

To limit the impact of between-subject variations in urine sampling conditions, we standardized biomarker concentrations using a two-step standardization method based on regression residuals. First, we estimated associations between ln-transformed biomarker concentrations and sampling conditions [hour of sampling (for EDEN only), gestational age at collection, duration of storage at room temperature before freezing, and season and day of sampling] using separate linear regression models for each biomarker adjusted for maternal age, body mass index (BMI) before pregnancy, parity, year of sampling, education, current occupation, active smoking, and center. Next, we used these data to estimate biomarker concentrations that would have been observed if all samples had been collected under the same conditions (e.g., 0730 hours for hour of sampling). Unless otherwise specified, all concentrations are the standardized values; we also report associations with biomarker concentrations not standardized for sampling conditions.

Associations between each standardized maternal urinary biomarker concentration and birth weight, birth length, or head circumference were estimated using separate weighted linear regressions. Biomarker concentrations (micrograms per liter) were ln-transformed or coded in tertiles. We performed tests of heterogeneity in outcome value across exposure tertiles; *p*-values of trend tests were estimated using categorical variables whose values corresponded to the tertile-specific median biomarker levels. Situations in which biomarkers exhibited heterogeneity in outcome across tertiles (low *p*-value, heterogeneity test) but with little support for a trend (high *p*-value, trend test) were considered as suggestive of a nonmonotonic association. [A monotonic trend does not reverse direction but may have flat segment (Rothman et al. 2008).] We also estimated adjusted relationships between standardized biomarker concentrations and birth weight using restricted cubic splines (Harrell 2001). Adjustment factors were all variables possibly related to birth outcomes (based on *a priori* knowledge), including maternal prepregnancy weight (broken stick model with a knot at 60 kg) (Slama and Werwatz 2005), maternal height (continuous), maternal smoking (never, 1–5, ≥ 6 cigarettes per day), parity (0, 1, ≥ 2), education level (high school or less, up to 2 years after high school, ≥ 3 years after high school), gestational duration (linear and quadratic terms), and recruitment center. In addition, we adjusted for urinary creatinine concentration (continuous, nontransformed) as a marker of urine dilution. Gestational duration was estimated using the date of last menstrual period (LMP) (Slama et al. 2008) or gestational duration assessed by the obstetrician if it differed from the LMP-based estimate by > 2 weeks. Adjustment for the obstetrician-estimated gestational age instead of the LMP-based gestational age did not modify results (not shown). Models for head circumference at birth were also adjusted for the mode of delivery (because passage through the birth canal may influence head circumference at birth).

We performed sensitivity analyses excluding women with pregnancy-induced hypertension (*n* = 17) or gestational diabetes (*n* = 15). All analyses were performed using STATA/SE, version 11 (StataCorp, College Station, TX, USA).

To draw our conclusion, we gave more weight to results in agreement with our *a priori* hypotheses, namely, an effect of BP3 and 2,5-DCP on male birth weight (Wolff et al. 2008). Other associations (highlighted on the basis of their *p*-values, without relying on threshold *p*-value to define statistical significance) were considered as hypothesis-generating.

## Results

*Study population.* The 287 women were 29 years old, on average; 17% smoked during the first trimester of pregnancy (Table 1). Average gestational age at delivery was 39.8 weeks, average birth weight was 3,393 g (5th–95th percentiles, 2,640–4,130 g) and seven newborns (2%) weighed < 2,500 g. Average birth length was 50 cm (5th–95th percentiles, 47–54 cm), and average head circumference at birth was 35 cm (5th–95th percentiles, 32–37 cm) (Table 1).

**Table 1:** Characteristics of French pregnant women and of their offspring (*n* = 287; EDEN and PELAGIE cohorts, 2002–2006).

Percentiles				
Characteristic	*n *(%) or mean*a*		5th	50th	95th
Maternal age (years) (mean)		29.3			22		29		38
Duration of gestation*b* (mean)		39.7			37		40		42
Birth weight (g) (mean)		3,393			2,640		3,390		4,130
Birth length (cm) (mean)		50.1			47		50		54
Head circumference at birth (cm) (mean)		34.7			32		35		37
Gestational age at sampling*b* (weeks) (mean)		21.7			9		26		28
Creatinine concentration (g/L) (mean)		1.2			0.4		1.1		2.2
Parity									
0		115	(40)						
1 previous child		114	(40)						
≥ 2 previous children		58	(20)						
Maternal education									
≤ High school		133	(47)						
High school + 2 years		62	(22)						
≥ High school + 3 years		86	(31)						
Missing value		6							
Active smoking									
0		238	(83)						
1–5 cigarettes/day		30	(11)						
≥ 6 cigarettes/day		17	(6)						
Missing value		2							
Prepregnancy BMI (kg/m^2^)									
< 18.5		29	(10)						
18.5–25.0		181	(64)						
> 25.0		73	(26)						
Missing value		4							
Hour of urinary sampling									
Before 0800 hours		108	(65)						
0800–1000 hours		40	(24)						
After 1000 hours		18	(11)						
Missing value		121							
**a**Sample size, unless otherwise specified. **b**Weeks of amenorrhea assessed by the date of the last menstrual period.									

We detected 8 of the 11 phthalate metabolites and 5 of the 9 phenols in at least 95% of the samples (Table 2).

**Table 2:** Urinary phenol (*n* = 191) and phthalate (*n* = 287) biomarker concentrations after correction for case–control sampling (EDEN and PELAGIE cohorts, 2002–2006).

Standardized concentrations,*a *percentiles (µg/L)					Measured concentrations, percentiles (µg/L)				
Analyte	LOD (µg/L)	% > LOD	5th	50th	95th	5th	50th	95th
Phenols																
2,4-DCP		0.2		95.9		0.2		0.8		8.6		0.2		0.9		10.2
2,5-DCP		0.2		99.5		1.4		6.4		316.0		1.8		10.2		442.0
BPA		0.4		98.5		0.8		3.1		10.1		0.6		2.7		9.8
BP3		0.4		80.5		0.2		1.3		74.5		0.3		1.7		143.0
TCS		2.3		84.1		1.0		17.5		464.6		1.6		24.1		634.0
MP		1.0		100.0		9.0		104.3		2689.7		9.1		97.8		3520
EP		1.0		67.7		0.2		1.5		38.2		0.7		4.1		62.3
PP		0.2		96.9		0.3		10.4		267.7		0.5		12.5		402.0
BP		0.2		79.5		0.1		2.2		63.6		0.1		1.7		53.8
Phthalates																
MEP		0.5		100.0		28.1		105.3		727.0		24.9		110.22		983.4
MBP		0.6		100.0		10.2		58.1		487.5		7.6		48.1		398.0
MiBP		0.3		100.0		15.6		64.7		365.3		10.9		45.9		219.0
MBzP		0.2		100.0		2.7		21.7		209.2		2.0		17.7		116.6
MCPP		0.2		98.3		0.6		3.2		13.8		0.4		2.2		10.0
MEHP		1.2		91.8		1.8		10.5		62.3		0.8		7.1		40.7
MEHHP		0.7		100		8.0		48.3		246.2		4.6		32.3		147.0
MEOHP		0.7		99.7		6.3		36.0		169.6		3.6		25.0		112.0
MECPP		0.6		100.0		18.9		67.2		303.0		11.6		43.8		183.0
MCOP		0.7		92.1		0.9		3.9		25.8		0.5		2.7		17.2
MCNP		0.6		91.8		0.9		3.1		22.8		0.6		1.7		11.7
Abbreviations: BP, butyl paraben; BPA, bisphenol A; BP3, benzophenone-3; EP, ethyl paraben; MBP, mono-*n*-butyl phthalate; MBzP, monobenzyl phthalate; MCNP, monocarboxy-isononyl phthalate; MCOP, monocarboxy-isooctyl phthalate; MCPP, mono(3-carboxypropyl) phthalate; MECPP, mono(2-ethyl-5-carboxypentyl) phthalate; MEHHP, mono(2-ethyl-5-hydroxyhexyl) phthalate; MEHP, mono(2-ethylhexyl) phthalate; MEOHP, mono(2-ethyl-5-oxohexyl) phthalate; MEP, monoethyl phthalate; MiBP, mono-isobutyl phthalate; MP, methyl paraben; PP, propyl paraben; TCS, Triclosan; 2,4-DCP, 2,4-dichlorophenol; 2,5-DCP, 2,5-dichlorophenol. **a**Concentrations were standardized for conditions of sampling such as hour of sampling (EDEN only), time elapsed between sample collections and freezing, season and day of sampling, and gestational age at collection.																

*Phenols and birth outcomes.* Birth weight decreased by 49 g [95% confidence interval (CI): –86, –13] in association with a 1-unit increase in ln-transformed 2,5-DCP concentration. After categorizing exposures in tertiles, boys in the highest exposure tertile were 152 g lighter, on average, compared with boys in the lowest tertile (95% CI: –299, –5) (Table 3). We observed a similar association between 2,4-DCP and birth weight [Table 3; see also Supplemental Material, Figure 1 (http://dx.doi.org/10.1289/ehp.1103634)], consistent with the high correlation between 2,4-DCP and 2,5-DCP (*r* = 0.95). 2,5-DCP ln-transformed concentration was also inversely associated with head circumference at birth (–0.1 cm, 95% CI: –0.2, 0.0).

**Table 3:** Adjusted associations between maternal urinary concentrations of phenol biomarkers standardized for sampling conditions*^a^* and birth outcomes (EDEN cohort, 2003–2006).

Analyte (µg/L)*a*	Change*b* in birth weight *n* = 191						Change*b* in birth length *n* = 190						Change*b* in head circumference *n* = 189					
	β (g) (95% CI)		*p*_het_*c*	*p*_trend_*d*	β (cm) (95% CI)		*p*_het_*c*	*p*_trend_*d*	β (cm) (95% CI)		*p*_het_*c*	*p*_trend_*d*
2,4-DCP																					
< 0.6		0			< 0.01		< 0.01		0			0.09		0.77		0			< 0.01		0.01
0.6–1.3		24	(–129, 177)						0.7	(0.1, 1.3)						0.8	(0.2, 1.4)				
≥ 1.3		–181	(–323, –40)						0.2	(–0.5, 0.9)						–0.3	(–0.8, 0.3)				
Ln(2,4-DCP)		–77	(–129, –25)						–0.1	(–0.4, 0.2)						–0.1	(–0.3, 0.1)				
2,5-DCP																					
< 3.9		0			0.10		0.03		0			0.53		0.70		0			0.14		0.10
3.9–13.9		–49	(–206, 108)						0.4	(–0.3, 1.0)						–0.3	(–0.8, 0.3)				
≥ 13.9		–152	(–299, –5)						0.3	(–0.4, 1.0)						–0.5	(–1.1, 0.0)				
Ln(2,5-DCP)		–49	(–86, –13)						0.0	(–0.2, 0.2)						–0.1	(–0.2, 0.0)				
BPA																					
< 2.2		0			0.10		0.70		0			0.98		0.83		0			0.04		0.01
2.2–4.7		169	(14, 324)						0.0	(–0.7, 0.8)						0.3	(–0.3, 0.9)				
≥ 4.7		85	(–62, 233)						0.1	(–0.7, 0.9)						0.8	(0.2, 1.3)				
Ln(BPA)		–9	(–98, 80)						0.0	(–0.4, 0.4)						0.3	(0.0, 0.7)				
BP3																					
< 0.7		0			0.20		0.09		0			0.17		0.14		0			0.12		0.04
0.7–2.7		–15	(–155, 124)						–0.3	(–1.0, 0.3)						0.2	(–0.5, 0.8)				
≥ 2.7		105	(–40, 250)						0.4	(–0.3, 1.0)						0.5	(0.0, 1.0)				
Ln(BP3)		26	(–2, 54)						0.1	(–0.1, 0.2)						0.1	(0.0, 0.2)				
TCS																					
< 4.5		0			0.68		0.79		0			0.68		0.59		0			0.31		0.18
4.5–51.3		–58	(–194, 78)						–0.2	(–0.9, 0.4)						0.2	(–0.3, 0.7)				
≥ 51.3		–40	(–171, 90)						0.1	(–0.6, 0.7)						–0.2	(–0.7, 0.3)				
Ln(TCS)		–6	(–31, 19)						0.0	(–0.1, 0.1)						–0.1	(–0.2, 0.0)				
MP																					
< 62.8		0			0.75		0.58		0			0.47		0.23		0			0.79		0.58
62.8–213.0		–32	(–168, 105)						0.2	(–0.5, 0.8)						0.2	(–0.4, 0.7)				
≥ 213.0		24	(–121, 168)						0.4	(–0.3, 1.2)						0.2	(–0.4, 0.8)				
Ln(MPB)		–2	(–37, 34)						0.1	(–0.1, 0.3)						0.0	(–0.1, 0.2)				
EP																					
< 0.6		0			0.98		0.86		0			0.24		0.97		0			0.47		0.94
0.6–3.7		10	(–129, 148)						0.6	(–0.1, 1.3)						0.4	(–0.2, 1.0)				
≥ 3.7		17	(–145, 178)						0.3	(–0.4, 0.9)						0.2	(–0.4, 0.8)				
Ln(EPB)		–1	(–43, 41)						0.1	(–0.1, 0.3)						0.1	(–0.1, 0.3)				
PP																					
< 4.7		0			1.00		0.95		0			0.98		0.93		0			0.52		0.62
4.7 – 24.8		–2	(–137, 132)						–0.1	(–0.7, 0.6)						–0.3	(–0.8, 0.2)				
≥ 24.8		–5	(–151, 140)						0.2	(–0.8, 0.7)						–0.2	(–0.8, 0.3)				
Ln(PPB)		–10	(–40, 19)						0.0	(–0.2, 0.2)						–0.1	(–0.2, 0.1)				
BP																					
< 0.6		0			0.97		0.95		0			0.58		0.49		0			0.61		0.45
0.6 – 6.8		–15	(–156, 126)						0.2	(–0.5, 1.0)						0.2	(–0.3, 0.7)				
≥ 6.8		–2	(–155, 150)						–0.1	(–0.9, 0.7)						0.3	(–0.3, 0.8)				
Ln(BPB)		–1	(–32, 29)						0.0	(–0.2, 0.2)						0.1	(–0.1, 0.2)				
∑PB (µmol/L)																					
< 0.5		0			0.95		0.86		0			0.61		0.38		0			0.80		0.66
0.5 – 1.6		–16	(–150, 118)						0.2	(–0.4, 0.9)						0.2	(–0.4, 0.7)				
≥ 1.6		6	(–143, 156)						0.4	(–0.4, 1.1)						0.2	(–0.4, 0.7)				
Ln(∑PB)		–3	(–39, 33)						0.1	(–0.1, 0.3)						0.0	(–0.2, 0.1)				
Abbreviations: BP, butyl paraben; BPA, bisphenol A; BP3, benzophenone-3; MP, methyl paraben; EP, ethyl paraben; PP, propyl paraben; TCS, Triclosan; 2,4-DCP, 2,4-dichlorophenol; 2,5-DCP, 2,5-dichlorophenol; ∑PB, molecular sum of parabens. Regression models were corrected for the overrepresentation of cases of malformations of the genitalia by a weighting approach. **a**Concentrations were standardized for conditions of sampling such as hour of sampling, time elapsed between sample collections and freezing, season and day of sampling, and gestational age at collection. **b**Adjusted for gestational duration, maternal prepregnancy weight and height, maternal smoking, maternal education level, parity, recruitment center, and creatinine level. Models for head circumference at birth were also adjusted for mode of delivery (cesarean section yes/no). **c***p*-Values of heterogeneity test. **d***p*-Values of monotonic trend test.																					

Each 1-unit increase in ln-transformed BP3 concentration was associated with an increase of 26 g in birth weight (95% CI: –2, 54) and of 0.1 cm in head circumference at birth (95% CI: 0.0, 0.2) (Table 3).

For BPA, estimates suggested an inverse U-shape association: birth weight increased by 169 g (95% CI: 14, 324) in the second BPA concentration tertile and by 85 g (95% CI: –62, 233) in the third concentration tertile, compared with the first [Table 3; see also Supplemental Material, Figure 2 (http://dx.doi.org/10.1289/ehp.1103634) for associations based on a restricted cubic spline model]. BPA concentrations were positively associated with head circumference, which increased by 0.8 cm in the highest BPA concentration tertile compared with the lowest tertile (95% CI: 0.2, 1.3) (Table 3).

For all other phenols, there was no evidence of associations with offspring measures at birth [all *p* for heterogeneity were > 0.24 (Table 3), and curves obtained using restricted cubic splines did not clearly support an association. See Supplemental Material, Figure 2 (http://dx.doi.org/10.1289/ehp.1103634)].

*Phthalates and birth outcomes.* For phthalate metabolite concentrations and birth weight, all *p* for trend were > 0.14 (Table 4). There was some evidence of heterogeneity in mean birth weight across concentration tertiles for some phthalate metabolites: the lowest *p*-values for heterogeneity were observed for mono(3-carboxypropyl) phthalate (MCPP) and MECPP; given the high values of *p* for trend, results were suggestive of nonmonotonic associations (Table 4). For MECPP, but less so for MCPP, this nonmonotonic association was also supported by the restricted cubic spline analysis to some extent [see Supplemental Material, Figure 3 (http://dx.doi.org/10.1289/ehp.1103634)].

**Table 4:** Adjusted associations between maternal urinary concentrations of phthalate biomarkers standardized for sampling conditions*a* and birth outcomes (EDEN and PELAGIE cohorts, 2002–2006).

Change*b* in birth weight *n* = 287						Change*b* in birth length *n* = 286						Change*b* in head circumference *n* = 285					
Analytes (µg/L)*a*	β (g) (95% CI)		*p*_het_*c*	*p*_trend_*d*	β (cm) (95% CI)		*p*_het_*c*	*p*_trend_*d*	β (cm) (95% CI)		*p*_het_*c*	*p*_trend_*d*
MEP																					
< 113.8		0			0.61		0.60		0			0.22		0.58		0			0.32		0.14
113.8–275.7		46	(–102, 194)						0.5	(–0.2, 1.1)						0.2	(–0.3, 0.7)				
≥ 275.7		–14	(–162, 133)						0.0	(–0.6, 0.7)						0.4	(–0.1, 1.0)				
Ln(MEP)		3	(–51, 57)						0.0	(–0.3, 0.2)						0.1	(–0.2, 0.3)				
MBP																					
< 45.6		0			0.44		0.42		0			0.69		0.91		0			0.89		0.63
45.6–85.5		52	(–101, 206)						0.3	(–0.4, 0.9)						0.1	(–0.5, 0.6)				
≥ 85.5		–30	(–174, 114)						0.1	(–0.6, 0.7)						0.1	(–0.4, 0.7)				
Ln(MBP)		–13	(–61, 35)						0.1	(–0.2, 0.3)						0.0	(–0.2, 0.2)				
MiBP																					
< 48.2		0			0.41		0.48		0			0.46		0.54		0			0.50		0.40
48.2–97.9		61	(–77, 200)						0.4	(–0.3, 1.1)						–0.1	(–0.6, 0.4)				
≥ 97.9		–31	(–190, 129)						0.3	(–0.4, 1.0)						0.2	(–0.5, 0.9)				
Ln(MiBP)		–44	(–110, 23)						0.0	(–0.3, 0.3)						–0.1	(–0.4, 0.1)				
MCPP																					
< 2.1		0			0.03		0.73		0			0.03		0.55		0			0.20		0.77
2.1–4.4		–198	(–343, –52)						–0.7	(–1.4, –0.1)						–0.5	(–0.1, 0.1)				
≥ 4.4		–95	(–243, 52)						–0.1	(–0.8, 0.7)						–0.3	(–0.9, 0.4)				
Ln(MCPP)		–34	(–91, 22)						0.1	(–0.2, 0.4)						–0.2	(–0.4, 0.1)				
MBzP																					
< 17.6		0			0.67		0.43		0			0.99		0.88		0			0.55		0.32
17.6–57.2		14	(–141, 170)						0.0	(–0.7, 0.7)						–0.2	(–0.8, 0.4)				
≥ 57.2		–50	(–223, 123)						0.1	(–0.9, 0.7)						–0.3	(–0.9, 0.3)				
Ln(MBzP)		–23	(–71, 24)						0.1	(–0.3, 0.2)						0.0	(–0.2, 0.2)				
MEHP																					
< 6.8		0			0.20		0.93		0			0.12		0.69		0			0.55		0.56
6.8–17.1		–122	(–261, 17)						–0.6	(–1.2, 0.0)						–0.2	(–0.7, 0.3)				
≥ 17.1		–37	(–184, 110)						–0.3	(–0.9, 0.3)						0.1	(–0.5, 0.6)				
Ln(MEHP)		1	(–60, 62)						0.0	(–0.3, 0.2)						0.0	(–0.2, 0.2)				
MEOHP																					
< 25.2		0			0.42		0.28		0			0.33		0.27		0			0.94		.75
25.2–56.8		–37	(–179, 105)						–0.2	(–0.8, 0.4)						0.0	(–0.5, 0.6)				
≥ 56.8		60	(–89, 209)						0.3	(–0.4, 1.0)						–0.1	(–0.6, 0.5)				
Ln(MEOHP)		5	(–56, 66)						0.1	(–0.2, 0.3)						0.0	(–0.3, 0.2)				
MEHHP																					
< 32.2		0			0.58		0.65		0			0.61		0.80		0			0.88		0.76
32.2–77.9		–60	(–202, 81)						–0.3	(–0.9, 0.4)						–1.0	(–0.6, 0.4)				
≥ 77.9		7	(–139, 154)						0.0	(–0.7, 0.7)						–0.1	(–0.7, 0.4)				
Ln(MEHHP)		4	(–54, 62)						0.1	(–0.2, 0.3)						0.0	(–0.2, 0.2)				
MECPP																					
< 45.8		0			0.08		0.59		0			0.40		0.43		0			0.90		0.65
45.8–105.4		–141	(–277, –5)						–0.3	(–1.0, 0.4)						0.0	(–0.5, 0.6)				
≥ 105.4		–20	(–162, 121)						0.2	(–0.6, 0.9)						0.1	(–0.4, 0.6)				
Ln(MECPP)		5	(–64, 73)						0.1	(–0.2, 0.4)						0.0	(–0.2, 0.3)				
MCOP																					
< 2.4		0			0.87		0.87		0			0.11		0.19		0			0.77		0.79
2.4–5.9		–40	(–192, 110)						–0.2	(–0.9, 0.4)						–0.1	(–0.7, 0.4)				
≥ 5.9		–27	(–200, 147)						0.4	(–0.5, 1.2)						0.0	(–0.6, 0.6)				
Ln(MCOP)		–8	(–72, 55)						0.1	(–0.2, 0.4)						0.0	(–0.2, 0.3)				
MCNP																					
< 2.3		0			0.85		0.99		0			0.11		0.08		0			0.89		0.72
2.3–4.6		–40	(–186, 107)						0.5	(–0.1, 1.2)						–0.1	(–0.7, 0.4)				
≥ 4.6		–15	(–189, 158)						0.9	(0.0, 1.7)						–0.1	(–0.8, 0.5)				
Ln(MCNP)		–3	(–67, 61)						0.3	(–0.1, 0.6)						–0.1	(–0.3, 0.1)				
DEHP (µmol/L)																					
< 0.4		0			0.43		0.29		0			0.18		0.39		0			0.80		0.68
0.4–0.9		–54	(–197, 88)						–0.4	(–1.0, 0.2)						0.1	(–0.3, 0.6)				
≥ 0.9		36	(–112, 186)						0.1	(–0.6, 0.9)						0.2	(–0.4, 0.7)				
Ln(DEHP)		5	(–60, 70)						0.1	(–0.2, 0.4)						0.0	(–0.2, 0.2)				
∑LMW (µmol/L)																					
< 1.2		0			0.32		0.14		0			0.29		0.53		0			0.12		0.52
1.2–2.7		–3	(–141, 135)						0.4	(–0.2, 1.1)						0.5	(0.0, 0.9)				
≥ 2.7		–100	(–248, 47)						0.0	(–0.7, 0.7)						0.4	(–0.3, 1.0)				
Ln(∑LMW)		–38	(–109, 34)						–0.1	(–0.4, 0.2)						–0.1	(–0.4, 0.2)				
∑HMW (µmol/L)																					
< 0.5		0			0.17		0.37		0			0.06		0.26		0			0.82		0.54
0.5–1.3		–99	(–243, 44)						–0.5	(–1.2, 0.1)						0.0	(–0.5, 0.5)				
≥ 1.3		19	(–161, 198)						0.2	(–0.6, 1.0)						0.2	(–0.5, 0.8)				
Ln(∑HMW)		–2	(–70, 65)						0.1	(–0.2, 0.4)						0.0	(–0.2, 0.2)				
Abbreviations: MBP, mono-*n*-butyl phthalate; MBzP, monobenzyl phthalate; MCNP, monocarboxyisononyl phthalate; MCOP, monocarboxy-isooctyl phthalate; MCPP, mono(3-carboxypropyl) phthalate; MECPP, mono(2-ethyl-5-carboxypentyl) phthalate; MEHP, mono(2-ethylhexyl) phthalate; MEHHP, mono(2-ethyl-5-hydroxyhexyl) phthalate; MEOHP, mono(2-ethyl-5-oxohexyl) phthalate; MEP, monoethyl phthalate; MiBP, mono-isobutyl phthalate. DEHP, molecular sum of 4 metabolites of di(2-ethylhexyl) phthalate (MEHP, MEHHP, MEOHP, MECPP); ∑LMW, molecular sum of low-MW phthalates (MEP, MBP, and MiBP); ∑HMW, molecular sum of high-MW phthalates (MBzP, MCPP, MEHP, MECPP, MEHHP, MEOHP, MCOP, and MCNP). Regression models were corrected for the overrepresentation of cases of malformations of the genitalia by a weighting approach. **a**Concentrations were standardized for conditions of sampling such as hour of sampling (EDEN only), time elapsed between sample collections and freezing, season and day of sampling, and gestational age at collection. **b**Adjusted for gestational duration, maternal prepregnancy weight and height, maternal smoking, maternal education level, parity, recruitment center, and urine dilution (creatinine level). Models for head circumference at birth were also adjusted for mode of delivery (cesarean section yes/no). **c***p*-Values of heterogeneity test. **d***p*-Values of monotonic trend test.																					

Regarding other birth outcomes, the lowest *p*-values for heterogeneity were observed with MCPP, monocarboxy-isooctyl phthalate (MCOP), monocarboxy-isononyl phthalate (MCNP), MEHP, and ΣHMW phthalates for birth length, and with ΣLMW phthalates for head circumference at birth (Table 4).

*Sensitivity analyses.* For phenols, associations with birth outcomes remained similar after exclusion of 48 cases of male malformations of the genitalia [See Supplemental Material, Table 1 (http://dx.doi.org/10.1289/ehp.1103634)]. Using biomarker concentrations not standardized for sampling conditions instead of concentrations standardized for sampling conditions did not markedly change associations between birth weight and, variously, 2,4-DCP, 2,5-DCP, BP3, or BPA. Similarly, associations between head circumference at birth and either BP3 or BPA remained similar (see Supplemental Material, Table 1).

In analyses restricted to controls only or using concentrations not standardized for sampling conditions, findings concerning phthalates were consistent with those obtained in the main analysis, except for MECPP: The birth weight change observed in the second MECPP concentration tertile was –141 g (95% CI: –277, –5) in the whole study population, –79 g (95% CI: –220, 61) after exclusion of cases, and –51 g (95% CI: –187, 84) using nonstandardized biomarker concentrations [see Supplemental Material, Table 2]. These sensitivity analyses are difficult to interpret; restriction to controls decreased population size, and standardization sometimes induced strong variations in the distribution of biomarker concentrations and hence in the cutoff values of tertiles.

Excluding women with pregnancy-induced hypertension or gestational diabetes did not modify our main results (data not shown).

## Discussion

Within our study population of male newborns, maternal urinary concentrations of 2,4-DCP and 2,5-DCP were associated with a birth weight decrease, whereas urinary concentrations of BP3 were positively associated with weight and head circumference at birth. BPA urinary concentrations were positively associated with head circumference. There was no evidence of monotonic associations between phthalate metabolite concentrations and birth weight.

*Phenols and birth outcomes.* In our study population, birth weight decreased by 152 g in the highest 2,5-DCP concentration tertile compared with the lowest tertile (95% CI: –299, –5). The only other human study addressing this issue reported a decrease by 210 g in the third 2,5-DCP concentration tertile compared with the first (95% CI: –348, –71) (Wolff et al. 2008). Concentrations of 2,5-DCP (and hence tertiles) were much higher in the study by Wolff et al. (median, 53 µg/L) than in ours (median standardized concentration, 6.4 µg/L). Wolff et al. also reported that boys were 0.3 cm shorter at birth per increase by 1 in ln(2,5-DCP) (95% CI: –0.6, –0.4). Our results did not clearly support such an association (0.0 cm per increase by 1 in ln(2,5-DCP); 95% CI: –0.2, 0.2), but birth length is not very accurately assessed, implying potentially strong measurement error. 2,5-DCP is a metabolite of 1,4-dichlorobenzene, which is used as chemical intermediate in the production of dyes and organic chemicals and found in mothballs and toilet-deodorizer blocks (Agency for Toxic Substances and Disease Registry 2006; Yoshida et al. 2002). Dichlorophenols may also be released from water treatments (Abrahamsson and Xie 1983).

2,4-DCP was also associated with a birth weight decrease. It is a metabolite of 1,3-dicholorobenzene, a minor contaminant of 1,4-dichlorobenzene (Agency for Toxic Substances and Disease Registry 2006; Yoshida et al. 2002), which may explain the high correlation reported between concentrations of both DCPs. 2,4-DCP is also an environmental transformation intermediate of the antiseptic agent triclosan and of herbicides such as 2,4-dichlorophenoxyacetic acid and 2-(2,4-dichlorophenoxy) propionic acid (Yang et al. 2010; Zona et al. 2002). Our results concerning 2,4-DCP are difficult to compare with those of Wolff et al. (2008), who studied male and female newborns altogether for this compound.

Taken together, these studies suggest an effect of dichlorophenols, or one of their precursors, on birth weight.

BP3 urinary concentrations were positively associated with weight and head circumference at birth. Boys were 105 g heavier (95% CI: –40, 250) in the highest BP3 concentration tertile compared with the lowest. Similarly, Wolff et al. (2008) noted a birth weight increase in male infants in the highest BP3 concentration tertile compared with the lowest (betas not reported); they did not report effect estimates for BP3 and head circumference in males. Exposure to BP3 likely results from use of consumer products as sunscreens or cosmetics (Calafat et al. 2008).

BPA urinary concentrations were positively associated with head circumference at birth. After categorizing exposures in tertiles, we observed an inverse U-shape association between BPA concentrations and birth weight. Such nonmonotonic dose–response curves between perinatal exposures to BPA and weight in early life have been reported in rodents (Rubin et al. 2001; Rubin and Soto 2009). However, urinary BPA concentrations were relatively low in our study population, enhancing the analytical uncertainties and hence the potential for exposure misclassification, which may limit our ability to distinguish monotonic from nonmonotonic associations.

*Phthalates and birth outcomes.* There was no strong evidence of monotonic association between phthalate metabolite concentrations and birth outcomes, except for the possible positive association between MCNP and birth length. Our analyses suggested nonmonotonic associations with birth weight and birth length for some phthalate metabolites. To our knowledge, such associations between phthalate metabolites and birth weight have not been reported previously in rodents or in humans and therefore should be considered cautiously.

*Study population.* To correct the overrepresentation of cases induced by our case–control design, we weighted the observations in regression models (Richardson et al. 2007). We also performed nonweighted analyses, restricted to controls, and our main results regarding birth weight remained similar to those based on the whole weighted study population.

*Exposure assessment.* Sampling conditions, such as hour of urine sampling or storage duration before freezing, may influence the concentrations of several biomarkers (Mahalingaiah et al. 2008; Samandar et al. 2009). We used a two-step standardization method based on regression residuals to reduce undesirable variability in biomarker urinary concentrations due to sampling conditions. To our knowledge, it is the first time that such an approach has been applied to study associations between phthalate or phenol prenatal exposures and birth outcomes. We repeated our analyses using concentrations not standardized for sampling conditions, and associations between DCPs or BP3 and birth weight or between BP3 and BPA and head circumference were similar.

*Limitations.* The assayed phenols and phthalates and their metabolites have relatively short half-lives in humans (typically ≤ 1 day), but accurate information is not available on half-lives in pregnant women, in whom metabolism may differ compared with nonpregnant women. We assessed exposures from the urinary concentrations at a single point during pregnancy; increasing the number of urine samples collected would have provided a more accurate estimate of the average exposure during the whole gestation. Adibi et al. (2008) reported that for phthalates, the probability of correctly classifying a woman into a low-exposure group based on a single urine sample, if she truly had low exposure based on multiple measurements, was between 0.43 for monoethyl phthalate (MEP) and 0.95 for mono-isobutyl phthalate (MiBP). Concerning phenols, BPA concentrations also vary during pregnancy (Braun et al. 2011). Therefore, clearly there is exposure misclassification, whose amplitude differs according to the biologically relevant exposure window (if any) and the compound considered.

We adjusted for many potential confounders, but residual confounding cannot be discarded. For example, specific metabolic disorders associated with both fetal growth and xenobiotic metabolism would constitute potential confounders. In our study, excluding women with pregnancy-induced hypertension or gestational diabetes did not alter associations with BPA and BP3 (not shown). Multiple comparisons are generally an issue in studies relying on several biomarkers. Although we did not formally correct for multiple comparisons, our choice to focus our conclusions on previously reported associations limits the risk of chance being a likely explanation for our main findings.

## Conclusions

In our study, there was no strong evidence of monotonic association between phthalate metabolite concentrations and birth outcomes. Urinary concentrations of 2,4-DCP and 2,5-DCP were negatively associated with birth weight, whereas BP3 concentrations were associated with a birth weight increase. Results concerning 2,5-DCP and BP3 are in agreement with another publication concerning male newborns from New York (Wolff et al. 2008).

## Supplemental Material

(172 KB) PDF
