# Supplementary material for: Exposure to Phthalates and Phenols during Pregnancy and Offspring Size at Birth
Source: Environ Health Perspect. 2011 Sep 7;120(3):464–70. doi: 10.1289/ehp.1103634 (PMC3295340; doi:10.1289/ehp.1103634)
Supplement: (172 KB) PDF [file ehp.1103634.s001.pdf]

# Supplemental Material

## Exposure to Phthalates and Phenols during Pregnancy and Offspring Size at Birth

Claire Philippat, Marion Mortamais, Cécile Chevrier, Claire Petit, Antonia M. Calafat, Xiaoyun Ye, Manori J. Silva, Christian Brambilla, Isabelle Pin, Marie-Aline Charles, Sylvaine Cordier, Rémy Slama

**Table of Contents**

Figure 1: Adjusted associations between 2,5-DCP (A), 2,4-DCP (B) and BP3 (C) maternal urinary concentrations (tertiles) standardized for sampling conditions, and birthweight (Eden cohort, n = 191). .......... 2

Figure 2: Birthweight as a function of phenol concentrations standardized for sampling conditions and coded as restricted cubic splines (log scale) (Eden cohort, 2003-2006). .......... 3

Figure 3: Birthweight as a function of phthalate concentrations standardized for sampling conditions and coded as restricted cubic splines (log scale) (Eden and Pélagie cohorts, 2002-2006) .......... 4

Table 1: Sensitivity analyses (phenols) - adjusted changes in weight and head circumference at birth associated with phenols urinary concentrations (Eden cohort, 2003-2006). .......... 5

Table 2: Sensitivity analyses (phthalates) - adjusted changes in weight and head circumference at birth associated with phthalate metabolites urinary concentrations (Eden and Pélagie cohorts, 2002-2006). .......... 7

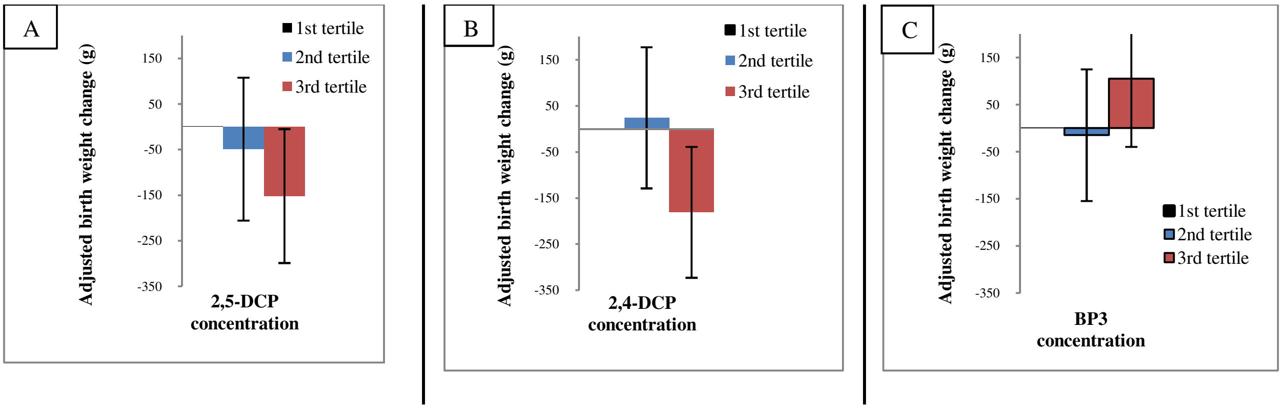


**Supplemental Material, Figure 1:** Adjusted[a] associations between 2,5-DCP (A), 2,4-DCP (B) and BP3 (C) maternal urinary concentrations (tertiles) standardized for sampling conditions, and birthweight (Eden cohort, n = 191).

Abbreviations: 2,4-DCP: 2,4-dichlorophenol, 2,5-DCP: 2,5-dichlorophenol, BP3: benzophenone 3.

[a] Adjusted for gestational duration, maternal pre-pregnancy weight and height, maternal smoking, maternal education level, parity, recruitment center and urine dilution (creatinine level).

Vertical bars indicate 95% confidence intervals.

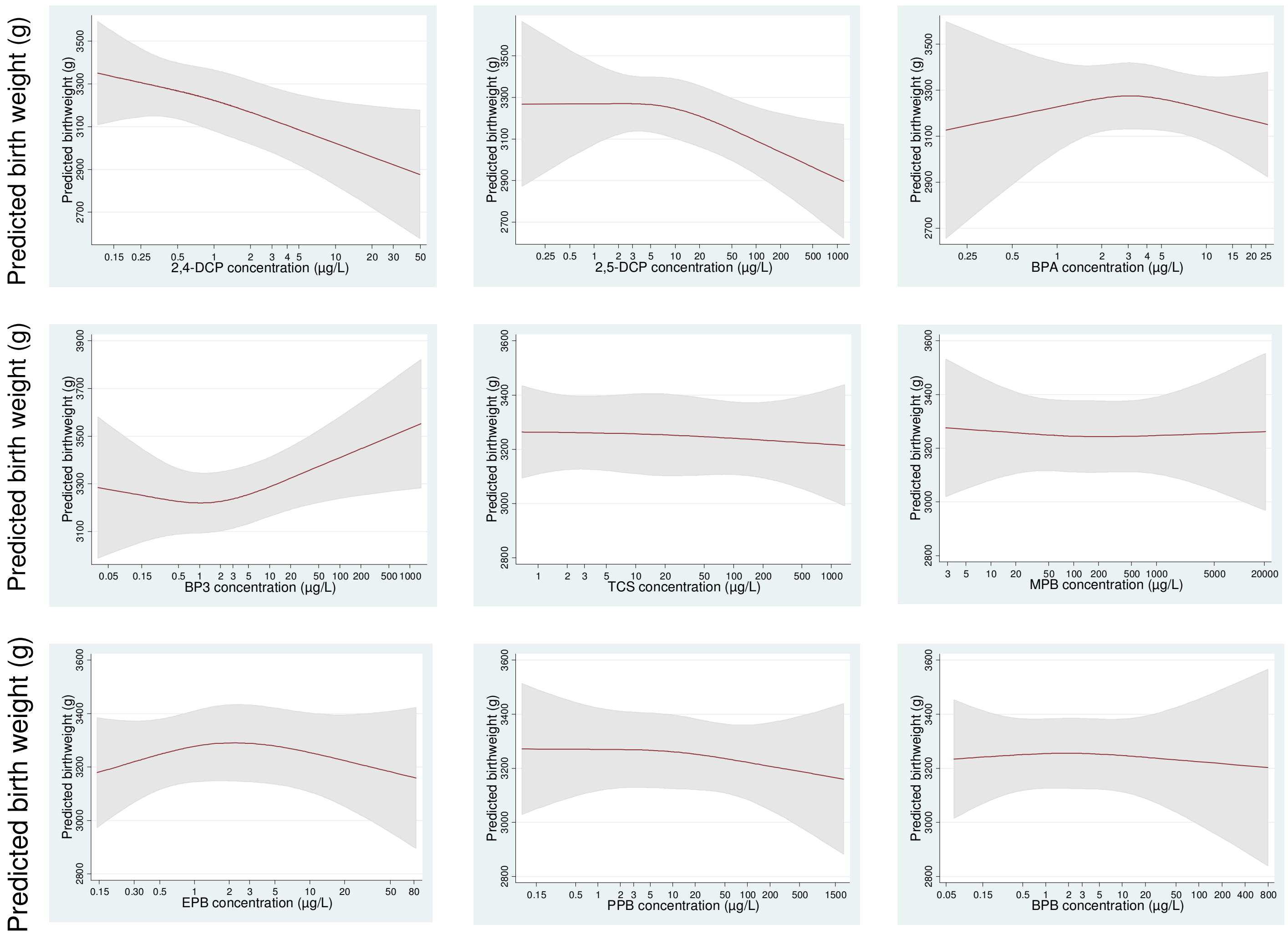


**Supplemental Material, Figure 2:** Birthweight as a function of phenol concentrations standardized for sampling conditions and coded as restricted cubic splines (log scale) (Eden cohort, 2003-2006).

The predicted curves are adjusted for gestational duration, maternal pre-pregnancy weight and height, maternal smoking, maternal education level, parity, recruitment center and creatinine concentration.
Abbreviations: BP: butyl paraben BPA: bisphenol A, BP3: benzophenone 3, MP: methyl paraben, EP: ethyl paraben, PP: propyl paraben, TCS: Triclosan, 2,4-DCP: 2,4-dichlorophenol, 2,5-DCP: 2,5-dichlorophenol.
Shading indicates 95% confidence interval.

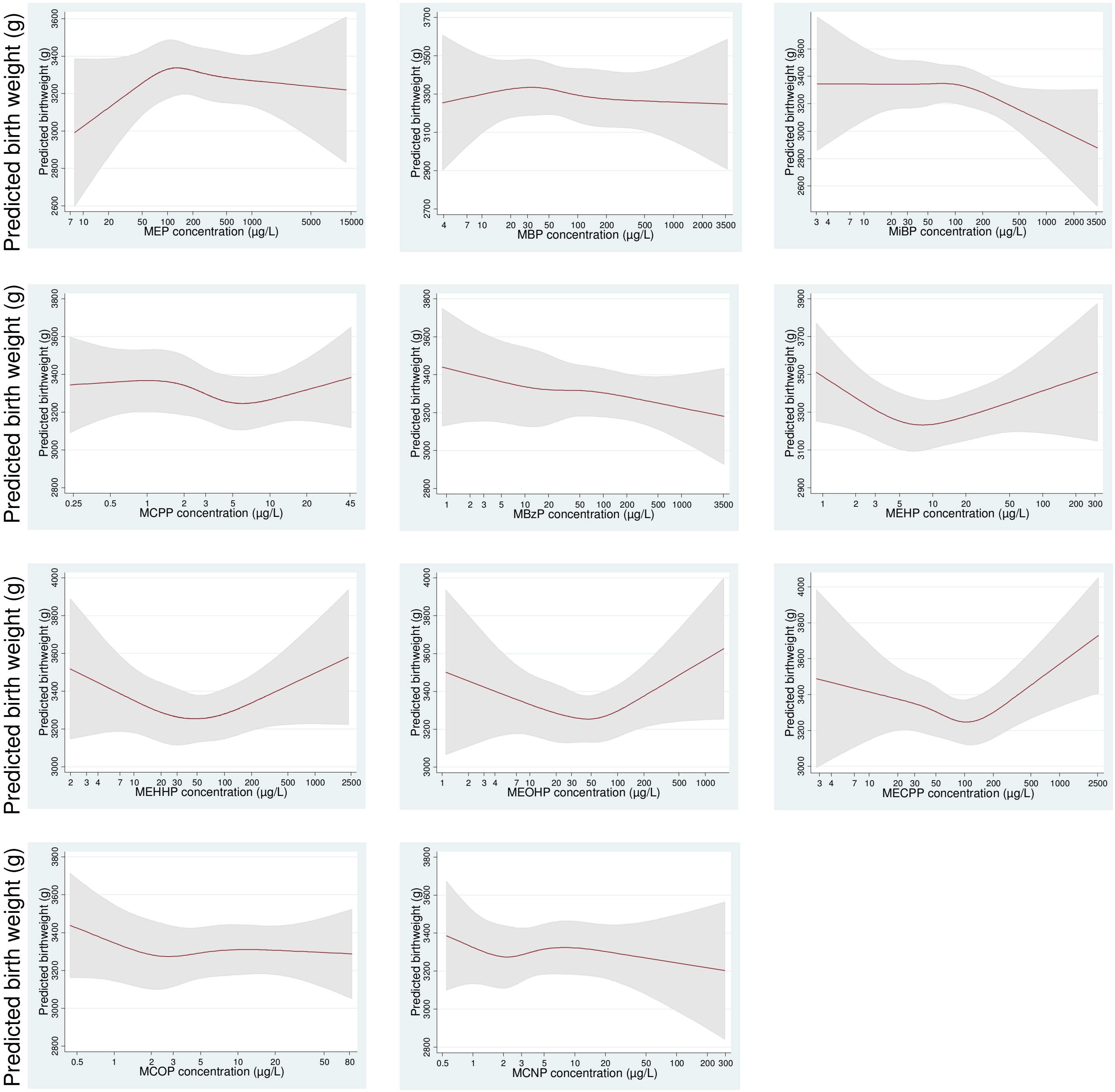


**Supplemental Material, Figure 3:** Birthweight as a function of phthalate concentrations standardized for sampling conditions and coded as restricted cubic splines (log scale) (Eden and Pélagie cohorts, 2002-2006).

The predicted curves are adjusted for gestational duration, maternal pre-pregnancy weight and height, maternal smoking, maternal education level, parity, recruitment center and creatinine concentration.
Shading indicates 95% confidence interval.
Abbreviations: MBP: mono-n-butyl phthalate, MBzP: monobenzyl phthalate, MCNP: monocarboxyisononyl phthalate, MCOP: monocarboxy-isooctyl phthalate, MCPP: mono(3-carboxypropyl) phthalate, MECPP: mono(2-ethyl-5-carboxypentyl) phthalate, MEHP: mono(2-ethylhexyl) phthalate, MEHHP: mono(2-ethyl-5-hydroxyhexyl) phthalate, MEOHP: mono(2-ethyl-5-oxohexyl) phthalate, MEP: monoethyl phthalate, MiBP: mono-isobutyl phthalate.

**Supplemental Material, Table 1:** Sensitivity analyses (phenols) - adjusted changes in weight and head circumference at birth associated with phenols urinary concentrations (Eden cohort, 2003-2006).

| Analyte (µg/L) | **Control group only (malformation cases excluded)[a]** | | | | | | **Non-standardized biomarker concentrations[b]** | | | | | |
|---|---|---|---|---|---|---|---|---|---|---|---|---|
| | Change in birthweight | | | Change in head circumference | | | Change in birthweight | | | Change in head circumference | | |
| | ß (g) | 95% CI | p | ß (cm) | 95% CI | p | ß (g) | 95% CI | p | ß (cm) | 95% CI | p |
| **2,4-DCP** | | | | | | | | | | | | |
| Tertile 1 | 0 | 0 | 0.01 [c] | 0 | 0 | 0.00 [c] | 0 | 0 | 0.09 [c] | 0 | 0 | 0.01 [c] |
| 2 | 22 | [-138; 183] | | 0.9 | [0.4; 1.5] | | -61 | [-221; 99] | | 0.4 | [-0.1; 0.9] | |
| 3 | -196 | [-360; -33] | | -0.2 | [-0.8; 0.4] | | -151 | [-291; 12] | | -0.4 | [-1.0; 0.1] | |
| Ln(2,4-DCP) | -84 | [-146; 23] | <0.01 [d] | -0.1 | [-0.3; 0.1] | 0.03 [d] | -68 | [-118; -19] | 0.03 [d] | -0.1 | [-0.2; 0.1] | 0.03 [d] |
| **2,5- DCP** | | | | | | | | | | | | |
| Tertile 1 | 0 | 0 | 0.12 [c] | 0 | 0 | 0.32 [c] | 0 | 0 | 0.34 [c] | 0 | 0 | 0.20 [c] |
| 2 | -56 | [-218; 105] | | -0.2 | [-0.9; 0.4] | | 32 | [-120; 184] | | 0.1 | [-0.5; 0.7] | |
| 3 | -172 | [-341; 3.8] | | -0.5 | [-1.1; 0.2] | | -73 | [-206; 60] | | -0.4 | [-0.9; 0.2] | |
| Ln(2,5-DCP) | -57 | [-100; 14] | 0.05 [d] | -0.1 | [-0.2; 0.1] | 0.16 [d] | -38 | [-71; -4] | 0.16 [d] | -0.1 | [-0.2; 0.0] | 0.08 [d] |
| **BPA** | | | | | | | | | | | | |
| Tertile 1 | 0 | 0 | 0.09 [c] | 0 | 0 | 0.11 [c] | 0 | 0 | 0.39 [c] | 0 | 0 | 0.12 [c] |
| 2 | 184 | [17; 352] | | 0.4 | [-0.3; 1.0] | | 101 | [-58; 259] | | 0.3 | [-0.3; 0.9] | |
| 3 | 91 | [-91; 274] | | 0.8 | [0.1;1.4] | | 21 | [-126; 168] | | 0.6 | [0.0; 1.2] | |
| Ln(BPA) | 5 | [-96; 105] | 0.75 [d] | 0.3 | [-0.1; 0.7] | 0.04 [d] | 5 | [-80; 90] | 0.86 [d] | 0.3 | [0.0; 0.6] | 0.04 [d] |
| **BP3** | | | | | | | | | | | | |
| Tertile 1 | 0 | 0 | 0.20 [c] | 0 | 0 | 0.25 [c] | 0 | 0 | 0.09 [c] | 0 | 0 | 0.22 [c] |
| 2 | -20 | [-184; 145] | | 0.2 | [-0.4; 0.8] | | -44 | [-196; 107] | | 0.0 | [-0.6; 0.6] | |
| 3 | 117 | [-46; 281] | | 0.5 | [-0.1;1.2] | | 107 | [-31; 246] | | 0.4 | [-0.1; 0.8] | |
| Ln(BP3) | 31 | [-5.6; 67] | 0.09 [d] | 0.1 | [0.0; 0.3] | 0.10 [d] | 35 | [7; 63] | 0.04 [d] | 0.1 | [0.0; 0.2] | 0.08 [d] |
| **TCS** | | | | | | | | | | | | |
| Tertile 1 | 0 | 0 | 0.65 [c] | 0 | 0 | 0.51 [c] | 0 | 0 | 0.84 [c] | 0 | 0 | 0.17 [c] |
| 2 | -74 | [-236; 86] | | 0.2 | [-0.4; 0.8] | | -38 | [-173; 96] | | 0.2 | [-0.3; 0.7] | |
| 3 | -22 | [-180; 136] | | -0.2 | [-0.8; 0.4] | | -27 | [-159; 106] | | -0.3 | [-0.8; 0.2] | |
| Ln(TCS) | -4 | [-36; 28] | 0.91 [d] | -0.1 | [-0.2; 0.1] | 0.35 [d] | -9 | [-34; 17] | 0.86 [d] | -0.1 | [-0.2; 0.0] | 0.09 [d] |
| **MPB** | | | | | | | | | | | | |
| Tertile 1 | 0 | 0 | 0.74 [c] | 0 | 0 | 0.88 [c] | 0 | | 0.66 [c] | 0 | 0 | 0.54 [c] |
| 2 | -34 | [-194; 124] | | 0.1 | [-0.5;0.7] | | 2 | [-138; 142] | | 0.3 | [-0.3; 0.8] | |
| 3 | 27 | [-140; 193] | | 0.2 | [-0.5; 0.8] | | 57 | [-79; 193] | | 0.3 | [-0.3; 0.8] | |
| **Ln(MPB)** | -1 | [-40; 39] | 0.58 [d] | 0.0 | [-0.2; 0.2] | 0.67 [d] | 5 | [-30; 41] | 0.36 [d] | 0.0 | [0.1; 0.2] | 0.50 [d] |

**Supplemental Material, Table 1 (continued)**

| Analyte (µg/L) | Control group only (malformation cases excluded)[a] | | | | | | Non-standardized biomarker concentrations[b] | | | | | |
|---|---|---|---|---|---|---|---|---|---|---|---|---|
| | Change in birthweight | | | Change in head circumference | | | Change in birthweight | | | Change in head circumference | | |
| | ß (g) | 95% CI | p | ß (cm) | 95% CI | p | ß (g) | 95% CI | p | ß (g) | 95% CI | p |
| **EPB** | | | | | | | | | | | | |
| Tertile 1 | 0 | 0 | 0.93 [d] | 0 | 0 | 0.46 [c] | 0 | 0 | 0.16 [c] | 0 | 0 | 0.23 [c] |
| 2 | 24 | [-149; 197] | | 0.4 | [-0.2; 1.0] | | 134 | [-5; 273] | | 0.5 | [-0.1; 1.1] | |
| 3 | 35 | [-147; 216] | | 0.2 | [-0.5; 0.9] | | 103 | [-52; 258] | | 0.4 | [-0.2; 1.0] | |
| Ln(EPB) | 3 | [-43; 49] | 0.76 | 0.1 | [-0.07; 0.28] | 0.98 [d] | 6 | [-34; 46] | 0.55 [d] | 0.1 | [-0.1; 0.3] | 0.54 [d] |
| **PPB** | | | | | | | | | | | | |
| Tertile 1 | 0 | 0 | 0.98 [d] | 0 | 0 | 0.48 [c] | 0 | 0 | 0.89 [c] | 0 | 0 | 0.78 [c] |
| 2 | -3 | [-167; 160] | | -0.4 | [-1.0; 0.3] | | -24 | [-170; 122] | | -0.1 | [-0.7; 0.4] | |
| 3 | -13 | [-179; 150] | | -0.3 | [-0.9; 0.3] | | 13 | [-124; 149] | | -0.2 | [-0.7; 0.4] | |
| Ln(PPB) | 38 | [-47; 21] | 0.86 | -0.1 | [-0.2; 0.1] | 0.57 [d] | -4 | [-33; 25] | 0.29 [d] | -0.1 | [-0.2; 0.1] | 0.58 [d] |
| **BPB** | | | | | | | | | | | | |
| Tertile 1 | 0 | 0 | 0.98 [d] | 0 | 0 | 0.71 [c] | 0 | 0 | 0.63 [c] | 0 | 0 | 0.65 [c] |
| 2 | 9 | [-156; 175] | | 0.2 | [-0.4; 0.8] | | 37 | [-108; 182] | | 0.1 | [-0.4;0.7] | |
| 3 | 15 | [-159; 190] | | 0.3 | [-0.4; 0.9] | | 65 | [-70; 201] | | 0.3 | [-0.3; 0.8] | |
| Ln(BPB) | 3 | [-34; 40] | 0.88 | 0.1 | [-0.1; 0.2] | 0.58 [d] | 9 | [-20; 39] | 0.41 [d] | 0.1 | [-0.1; 0.2] | 0.41 [d] |
| **∑PB** (µmol/L) | | | | | | | | | | | | |
| Tertile 1 | 0 | 0 | 0.95 [d] | 0 | 0 | 0.89 [c] | 0 | 0 | 0.72 [c] | 0 | 0 | 0.67 [c] |
| 2 | -18 | [-178; 141] | | 0.1 | [-0.5; 0.7] | | 4 | [-131; 139] | | 0.1 | [-0.4; 0.7] | |
| 3 | 7 | [-160; 175] | | 0.1 | [-0.5; 0.8] | | 53 | [-84; 191] | | 0.3 | [-0.3; 0.8] | |
| Ln(∑PB) | -2 | [-42; 38] | 0.85 | 0.0 | [-0.2; 0.1] | 0.77 [d] | 5 | [-31; 41] | 0.42 [d] | 0.0 | [-0.1; 0.2] | 0.40 [d] |

Abbreviations: BPA: bisphenol A, BP3: benzophenone 3, TCS: Triclosan, 2,4-DCP: 2,4-dichlorophenol, 2,5-DCP: 2,5-dichlorophenol, MP: methyl paraben, EP: ethyl paraben, PP: propyl paraben, BP: butyl paraben, ∑PB: molecular sum of parabens. Adjustment factors were maternal pre-pregnancy weight and height, maternal smoking, maternal education level, parity, recruitment center and creatinine level. Models for head circumference were further adjusted for mode of delivery (cesarean section yes/no).

[a] Non-weighted analyses restricted to controls; concentrations were standardized for conditions of sampling; the limits of exposure tertiles were the same as those defined in the whole population (as in table 3), n=143.

[b] Weighted analyses in the whole population, non-standardized concentrations; n=191.

[c] p-values of heterogeneity test.

[d] p-values of monotonic trend test.

Results of the sensitivity analyses for birthlength are available from the corresponding author.

**Supplemental Material, Table 2:** Sensitivity analyses (phthalates) - adjusted changes in weight and head circumference at birth associated with phthalate metabolites urinary concentrations (Eden and Pélagie cohorts, 2002-2006).

| Analyte (ug/L) | **Control group only (malformation cases excluded)[a]** | | | | | | **Non-standardized biomarker concentrations[b]** | | | | | |
|---|---|---|---|---|---|---|---|---|---|---|---|---|
| | Change in birthweight | | | Change in head circumference | | | Change in birthweight | | | Change in head circumference | | |
| | ß (g) | 95% CI | p | ß (cm) | 95% CI | p | ß (g) | 95% CI | p | ß (cm) | 95% CI | p |
| **MEP** | | | | | | | | | | | | |
| Tertile 1 | 0 | 0 | 0.42[c] | 0 | 0 | 0.15[c] | 0 | 0 | 0.60[c] | 0 | 0 | 0.21[c] |
| 2 | 78 | [-56; 213] | | 0.3 | [-0.2; 0.8] | | 62 | [-72; 196] | | 0.4 | [-0.1; 1.0] | |
| 3 | 8 | [-130; 147] | | 0.5 | [0.0; 1.0] | | 57 | [-80; 195] | | 0.3 | [-0.1; 0.8] | |
| Ln(MEP) | 0 | [-54; 54] | 0.70[d] | 0.1 | [-0.1; 0.3] | 0.07[d] | 14 | [-39; 67] | 0.52[d] | 0.0 | [-0.2; 0.2] | 0.33[d] |
| **MBP** | | | | | | | | | | | | |
| Tertile 1 | 0 | 0 | 0.33[c] | 0 | 0 | 0.74[c] | 0 | 0 | 0.65[c] | 0 | 0 | 0.42[c] |
| 2 | 73 | [-69; 217] | | 0.2 | [-0.4; 0.7] | | 36 | [-112; 185] | | 0.1 | [-0.3; 0.6] | |
| 3 | -16 | [-162; 130] | | 0.2 | [-0.3; 0.8] | | 72 | [-81; 225] | | 0.4 | [-0.2; 0.9] | |
| Ln(MBP) | -8 | [-63; 47] | 0.40[d] | 0.0 | [-0.2; 0.2] | 0.50[d] | 6 | [-42; 54] | 0.37[d] | 0.0 | [-0.2; 0.2] | 0.19[d] |
| **MiBP** | | | | | | | | | | | | |
| Tertile 1 | 0 | 0 | 0.25[c] | 0 | 0 | 0.80[c] | 0 | 0 | 0.81[c] | 0 | 0 | 0.76[c] |
| 2 | 30 | [-114; 174] | | -0.2 | [-0.7; 0.4] | | -33 | [-158; 93] | | 0.0 | [-0.5; 0.4] | |
| 3 | -80 | [-229; 70] | | -0.1 | [-0.7; 0.5] | | -44 | [-189; 100] | | 0.2 | [-0.4; 0.8] | |
| Ln(MiBP) | -60 | [-125; 5] | 0.14[d] | -0.2 | [-0.5; 0.0] | 0.98[d] | -29 | [-99; 42] | 0.61[d] | -0.1 | [-0.3; 0.2] | 0.47[d] |
| **MCPP** | | | | | | | | | | | | |
| Tertile 1 | 0 | 0 | 0.10[c] | 0 | 0 | 0.23[c] | 0 | 0 | 0.24[c] | 0 | 0 | 0.51[c] |
| 2 | -159 | [-303; -15] | | -0.5 | [-1.0; 0.1] | | -101 | [-234; -31] | | -0.3 | [-0.7; 0.2] | |
| 3 | -104 | [-252; 43] | | -0.4 | [-1.0; 0.2] | | -13 | [-155; 129] | | -0.3 | [-0.9; 0.3] | |
| Ln(MCPP) | -27 | [-92; 37] | 0.59[d] | -0.1 | [-0.4; 0.1] | 0.49[d] | -9 | [-66; 49] | 0.76[d] | -0.1 | [-0.3; 0.1] | 0.47[d] |
| **MBzP** | | | | | | | | | | | | |
| Tertile 1 | 0 | 0 | 0.67[c] | 0 | 0 | 0.32[c] | 0 | 0 | 0.58[c] | 0 | 0 | 0.34[c] |
| 2 | 31 | [-120; 182] | | -0.1 | [-0.7; 0.5] | | 75 | [-66; 216] | | -0.3 | [-0.8; 0.2] | |
| 3 | -30 | [-198; 139] | | -0.4 | [-1.1; 0.2] | | 44 | [-104; 192] | | 0.0 | [-0.6; 0.7] | |
| Ln(MBzP) | -29 | [-82; 24] | 0.47[d] | -0.1 | [-0.3; 0.1] | 0.13[d] | -12 | [-57; 32] | 0.74[d] | 0.0 | [-0.2; 0.2] | 0.66[d] |
| **MEHP** | | | | | | | | | | | | |
| Tertile 1 | 0 | 0 | 0.56[c] | 0 | 0 | 0.60[c] | 0 | 0 | 0.11[c] | 0 | 0 | 0.34[c] |
| 2 | -76 | [-213; 62] | | -0.3 | [0.8; 0.3] | | -124 | [-258; 10] | | -0.2 | [-0.7; 0.3] | |
| 3 | -51 | [-195; 92] | | -0.1 | [-0.7; 0.4] | | -10 | [-176; 155] | | 0.2 | [-0.3; 0.8] | |
| Ln(MEHP) | -17 | [-72; 38] | 0.71[d] | 0.0 | [-0.2; 0.2] | 0.87[d] | 11 | [-53; 75] | 0.78[d] | 0.1 | [-0.1; 0.3] | 0.23[d] |

**Supplemental Material, Table 2 (continued)**

| Analyte (ug/L) | Control group only (malformation cases excluded)[a] | | | | | | Non-standardized biomarker concentrations[b] | | | | | |
|---|---|---|---|---|---|---|---|---|---|---|---|---|
| | Change in birthweight | | | Change in head circumference | | | Change in birthweight | | | Change in head circumference | | |
| | ß (g) | 95% CI | p | ß (cm) | 95% CI | p | ß (g) | 95% CI | p | ß (cm) | 95% CI | p |
| **MEOHP** | | | | | | | | | | | | |
| Tertile 1 | 0 | 0 | 0.95 [c] | 0 | 0 | 0.36 [c] | 0 | 0 | 0.44 [c] | 0 | 0 | 0.06 [c] |
| 2 | 10 | [-130; 151] | | -0.2 | [-0.3; 0.8] | | -84 | [-223; 56] | | -0.6 | [-1.1; -0.1] | |
| 3 | -12 | [-154; 131] | | -0.1 | [-0.7; 0.4] | | -86 | [-248; 76] | | -0.4 | [-1.1; 0.3] | |
| Ln(MEOHP) | -19 | [-78; 39] | 0.81[d] | -0.1 | [-0.3; 0.2] | 0.38[d] | 15 | [-49;80] | 0.41[d] | 0.0 | [-0.2; 0.3] | 0.55[d] |
| **MEHHP** | | | | | | | | | | | | |
| Tertile 1 | 0 | 0 | 0.80 [c] | 0 | 0 | 0.60 [c] | 0 | 0 | 0.19 [c] | 0 | 0 | 0.87 [c] |
| 2 | -10 | [-151; 131] | | 0.1 | [-0.5; 0.6] | | -128 | [-275; 20] | | -0.1 | [-0.6; 0.4] | |
| 3 | -45 | [-189; 99] | | -0.2 | [-0.7; 0.4] | | -25 | [-181; 131] | | -0.1 | [-0.7; 0.5] | |
| Ln(MEHHP) | -19 | [-77; 38] | 0.50[d] | -0.1 | [-0.3; 0.2] | 0.36[d] | 10 | [-51; 71] | 0.80[d] | 0.0 | [-0.2; 0.2] | 0.89[d] |
| **MECPP** | | | | | | | | | | | | |
| Tertile 1 | 0 | 0 | 0.53 [c] | 0 | 0 | 0.99 [c] | 0 | 0 | 0.73 [c] | 0 | 0 | 0.64 [c] |
| 2 | -79 | [-220; 61] | | 0.0 | [-0.5; 0.6] | | -51 | [-187; 84] | | -0.2 | [-0.8; 0.3] | |
| 3 | -38 | [-184; 107] | | 0.0 | [-0.5; 0.6] | | -50 | [-213; 113] | | 0.0 | [-0.6; 0.5] | |
| Ln(MECPP) | -23 | [-88; 42] | 0.92[d] | -0.1 | [-0.3; 0.2] | 0.90[d] | 16 | [-57; 88] | 0.66[d] | 0.1 | [-0.2; 0.3] | 0.79[d] |
| **MCOP** | | | | | | | | | | | | |
| Tertile 1 | 0 | 0 | 0.65 [c] | 0 | 0 | 0.94 [c] | 0 | 0 | 0.16 [c] | 0 | 0 | 0.49 [c] |
| 2 | -68 | [-213; 77] | | -0.1 | [-0.6; 0.5] | | -106 | [-251; 38] | | 0.0 | [-0.5; 0.5] | |
| 3 | -56 | [-214; 102] | | 0.0 | [-0.6; 0.6] | | -1 | [-161; 160] | | 0.3 | [-0.3; 0.8] | |
| Ln(MCOP) | -32 | [-94; 29] | 0.65[d] | 0.0 | [-0.2; 0.3] | 0.88[d] | 12 | [-55; 79] | 0.55[d] | 0.1 | [-0.1; 0.3] | 0.28[d] |
| **MCNP** | | | | | | | | | | | | |
| Tertile 1 | 0 | 0 | 0.92 [c] | 0 | 0 | 0.86 [c] | 0 | 0 | 0.28 [c] | 0 | 0 | 0.24 [c] |
| 2 | -13 | [-153; 127] | | 0.1 | [-0.5; 0.6] | | -64 | [-204; 77] | | -0.4 | [-0.9; 0.1] | |
| 3 | -28 | [-170; 113] | | -0.1 | [-0.6; 0.5] | | 46 | [-109; 202] | | 0.2 | [-0.9; 0.4] | |
| Ln(MCNP) | -14 | [-71; 43] | 0.70[d] | -0.1 | [-0.3; 0.1] | 0.70[d] | 12 | [-51; 75] | 0.44[d] | -0.1 | [-0.3; 0.2] | 0.57[d] |
| **DEHP** (µmol/L) | | | | | | | | | | | | |
| Tertile 1 | 0 | 0 | 0.92 [c] | 0 | 0 | 0.66 [c] | 0 | 0 | 0.60 [c] | 0 | 0 | 0.67 [c] |
| 2 | -30 | [-169; 110] | | 0.2 | [-0.3; 0.7] | | -71 | [-209; 67] | | -0.2 | [-0.8; 0.3] | |
| 3 | -19 | [-164; 126] | | 0.0 | [-0.3; 0.7] | | -34 | [-190; 122] | | -0.1 | [-0.6; 0.5] | |
| Ln(DEHP) | -21 | [-83; 40] | 0.89[d] | -0.1 | [-0.3; 0.2] | 0.75[d] | 15 | [-53; 84] | 0.89[d] | 0.0 | [-0.2; 0.3] | 0.98[d] |

**Supplemental Material, Table 2 (continued)**

| Analyte (ug/L) | Control group only (malformation cases excluded)[a] | | | | | | Non-standardized biomarker concentrations[b] | | | | | |
|---|---|---|---|---|---|---|---|---|---|---|---|---|
| | Change in birthweight | | | Change in head circumference | | | Change in birthweight | | | Change in head circumference | | |
| | ß (g) | 95% CI | p | ß (cm) | 95% CI | p | ß (g) | 95% CI | p | ß (cm) | 95% CI | p |
| **∑LMW** (µmol/L) | | | | | | | | | | | | |
| Tertile 1 | 0 | 0 | 0.51[c] | 0 | 0 | 0.24[c] | 0 | 0 | 0.61[c] | 0 | 0 | 0.63[c] |
| 2 | -2 | [-151; 146] | | 0.5 | [-0.1; 1.0] | | 34 | [-101; 167] | | -0.1 | [-0.6; 0.4] | |
| 3 | -76 | [-238; 86] | | 0.4 | [-0.2;1.0] | | -36 | [-183; 111] | | 0.2 | [-0.4; 0.8] | |
| Ln(∑LMW) | -41 | [-114; 33] | 0.26[d] | -0.1 | [-0.4; 0.2] | 0.57[d] | -5 | [-72; 61] | 0.50[d] | -0.1 | [-0.3; 0.2] | 0.44[d] |
| **∑HMW** (µmol/L) | | | | | | | | | | | | |
| Tertile 1 | 0 | 0 | 0.84[c] | 0 | 0 | 0.97[c] | 0 | 0 | 0.12[c] | 0 | 0 | 0.37[c] |
| 2 | -42 | [-185; 101] | | 0.1 | [-0.5; 0.6] | | -141 | [-281; -1] | | -0.2 | [-0.8; 0.3] | |
| 3 | -24 | [-182; 133] | | 0.0 | [-0.6; 0.6] | | -56 | [-213; 101] | | 0.1 | [-0.4; 0.7] | |
| Ln(∑HMW) | -34 | [-98; 31] | 0.93[d] | -0.1 | [-0.3; 0.2] | 0.99[d] | 9 | [-60; 79] | 0.84[d] | 0.1 | [-0.2; 0.3] | 0.40[d] |

Abbreviations: MEP: monoethyl phthalate, MBP: mono-n-butyl phthalate, MiBP: mono-isobutyl phthalate, MBzP: monobenzyl phthalate, MCPP: mono(3-carboxypropyl) phthalate, MEHP: mono(2-ethylhexyl) phthalate, MEHHP: mono(2-ethyl-5-hydroxyhexyl) phthalate, MEOHP: mono(2-ethyl-5-oxohexyl) phthalate, MECPP: mono(2-ethyl-5-carboxypentyl) phthalate, MCOP: monocarboxy-isooctyl phthalate, MCNP: monocarboxyisononyl phthalate, DEHP: molecular sum of 4 metabolites of di(2-ethylhexyl) phthalate, ∑LMW: molecular sum of low molecular weight phthalates, ∑HMW: molecular sum of high molecular weight phthalates.
Adjustment factors were maternal pre-pregnancy weight and height, maternal smoking, maternal education level, parity, recruitment center and creatinine level. Models for head circumference were further adjusted for mode of delivery (cesarean section yes/no).

[a] Non-weighted analyses restricted to controls, concentrations were standardized for conditions of sampling; the limits of exposure tertiles were the same as those defined in the whole population (as in table 4); n=215.

[b] Weighted analyses in the whole population, non-standardized concentrations; n=287.

[c] p-values of heterogeneity test.

[d] p-values of monotonic trend test.

Results of the sensitivity analyses for birth length are available from the corresponding author.
